# The role of prenatal choline and its impact on neurodevelopmental disorders

**DOI:** 10.3389/fnut.2024.1463983

**Published:** 2024-11-18

**Authors:** María F. Mujica-Coopman, Evan M. Paules, Isis Trujillo-Gonzalez

**Affiliations:** ^1^Institute of Nutrition and Food Technology (INTA), University of Chile, Santiago, Chile; ^2^Nutrition Research Institute, University of North Carolina at Chapel Hill, Kannapolis, NC, United States; ^3^Department of Nutrition, Gillings School of Global Public Health, University of North Carolina at Chapel Hill, Chapel Hill, NC, United States

**Keywords:** choline, Rett syndrome, MeCP2, fragile X, neurodevelopment, down syndrome

## Abstract

Extensive evidence demonstrates that prenatal nutrition is crucial for adequate fetal development. Specifically, maternal choline intake plays a significant role in gene expression, epigenetics, and cell membrane formation. Preclinical models have shown that maternal dietary intake improves the development of the cerebral cortex and hippocampus. This review focuses on the role of prenatal choline intake and discusses its potential role in neurodevelopmental disorders especially since choline has emerged as a promising coadjutant to mitigate cognitive and developmental disorders. However, more evidence regarding timing, dosage, and molecular mechanisms is needed. In this review, we discuss the impact of prenatal choline availability, evidence from current models, and gaps to address in how choline may impact the symptomology and manifestation of Rett syndrome, fragile X syndrome, and Down syndrome. Our ultimate goal is to highlight the importance of choline for maternal health and its potential beneficial impact on neurodevelopmental disorders.

## 1 Introduction

The periconceptional and prenatal periods are considered among the most critical and vulnerable stages of life, mainly because of their profound impact on brain development and long-term health outcomes (1). Brain development, including processes such as network maturation, gamma-aminobutyric acid (GABA) function, and neuronal migration, as well as cognitive abilities, is largely established during pregnancy and early on in postnatal development. As a result, the risk of neurodevelopmental disorders is increased during this period (2).

Nutrition factors are paramount during the prenatal window for optimal retinal, brain, and cognitive development.

Among key prenatal nutrients, choline is an essential nutrient for fetal and infant development. Choline and its metabolites are critical for cell membrane formation, gene expression, cell signaling, and neurotransmission (3). In addition, choline has a key role in one-carbon metabolism as a precursor of betaine, a methyl donor for methylation reactions such as DNA and histone methylation. These methylation processes are well-known epigenetic mechanisms that influence fetal growth and development programming (4). In adults, choline also contributes to the homeostasis of liver, muscle, and cellular membrane structure (5, 6). Although choline can be made from *de novo* synthesis through phosphatidylethanolamine N-methyl transferase (PEMT), this is not sufficient to meet the cellular functions requiring dietary consumption of choline (5, 6).

According to the National Academy of Medicine (NAM) in the United States, dietary reference intake values are 550 mg of choline/day and 425 mg of choline/day for male and female adults, respectively (7, 8). The choline requirement increases during pregnancy to 450 mg/day (9) and during lactation to 550 mg/day, ensuring the health of the infant and mother (9). However, >90% of pregnant women in the United States do not meet dietary intake requirements for choline (10, 11).

Neurodevelopmental disorders (NDD) are a group of disorders that affect the development and function of the brain and nervous systems. Typically, these disorders are evident in early childhood and persist throughout an individual’s life. Some of these disorders, with a known genetic origin, will be covered by this review, including Rett syndrome (RTT), fragile X syndrome (FXS), and trisomy 21 (Down syndrome, DS).

Globally, Rett syndrome has an estimated prevalence of 7.1 per 100,000 females and is typically lethal in males who do not survive past infancy (12, 13). Molecular genetic testing (14) has shown that FXS has an estimated prevalence of 16–25 per 100,000 males, with approximately half that prevalence in females. On the other hand, DS has a prevalence of 57 cases per 100,000 live births in the United States (15, 16). The prevalence rates are expressed as cases per live births (17). Currently, there are no known treatments for these challenging disorders, which results in a lifelong need for medical, therapeutic services, and pharmacological needs. In the United States, according to the National Center on Birth Defects and Developmental Disabilities, the median cost for individuals with DS is 12–13 times higher than for children without it (18); for FXS, the all-caused direct cost was 2.5 times higher in FXS individuals than in non-FXS individuals (19). Strikingly, the average of medical expenditures for individuals with Rett Syndrome was calculated at $40,326 yearly, per patient (13). Given these challenges, identifying accessible and cost-effective therapies that can ameliorate symptomology for individuals with these NDDs is of critical importance.

In this review, we will explore the critical role of choline metabolism in brain development, with a particular focus on molecular mechanisms and neurogenesis. In addition, we will examine the abovementioned NDDs, offering an overview of their clinical features and the molecular mechanisms driving their pathology. Our goal is to underscore the importance of adequate intake of choline during pregnancy and to discuss the potential of this essential micronutrient in improving neurological outcomes in these complex conditions.

## 2 The key role of prenatal choline in optimal brain development during pregnancy

According to Barker’s fetal programming theory, established in the 1950s, and later expanded to the Developmental Origins of Health and Disease (DOHaD) theory, environmental and lifestyle factors such as dietary intake and nutritional status in critical periods of development, such as pregnancy, can have long-term health consequences in the offspring later in life (20, 21). Pregnancy is a vulnerable period of life due to the increased rate of cellular differentiation and proliferation (22), where the nutritional status of the mother plays a crucial role in ensuring a healthy pregnancy and optimal fetal brain development (23). In this sense, adequate maternal nutrition is critical to support offspring growth with brain development being a critical organ to support during this period.

During mammalian neurogenesis, the developing brain requires a steady supply of energy and nutrients to support cellular differentiation and structural formation. After conception, neural progenitor cells (NPCs) undergo self-renewal and several cycles of division, leading to differentiation into neurons, astrocytes, or oligodendrocytes, and migration to construct the cortical layers of the cerebral cortex (24). Efforts have been made to understand how maternal nutrition influences development from embryogenesis through postnatal stages.

For instance, low choline intake during pregnancy significantly alters progenitor cell fate, altering proliferation and differentiation. Suboptimal choline availability can disrupt communication between brain regions (cholinergic neurotransmission), potentially leading to altered behavior (25, 26). In a controlled feeding trial, infants whose mothers consumed 930 mg of choline per day during the third trimester of pregnancy were compared to those whose mothers consumed 450 mg per day. The infants were tested for information processing speed and visuospatial memory at 4, 7, 10, and 13 months of age, by measuring the latency of saccadic eye movements. Notably, the mean reaction time across all four ages was faster for infants whose mothers had higher choline intake (27). These findings suggest that the third trimester of pregnancy may be a critical period during which maternal choline influences cognitive development, particularly in terms of processing speed.

The developing brain requires nutrients to be “just in time” to avoid disruptions in developmental stages, ensuring healthy brain structure. However, under conditions of low choline availability, these stages, particularly neurogenesis, may be altered. This process occurs within the third trimester of human pregnancy or between embryonic day (E) 11.5 to E17.5 in rodents. While low choline availability has many consequences across the hippocampus, the center of memory, and the cerebral cortex, the center of executive function, the exact molecular mechanism by which choline deficiency elicits these effects remains under investigation. However, mechanisms such as the regulation of microRNAs and the Salvador–Warts–Hippo pathway (also known as Hippo signaling pathway), which govern cellular proliferation and apoptosis, have been identified as potential contributors (28, 29). Low choline intake during pregnancy not only alters brain structure (cerebral cortex layering) but also affects behavior later in life (30).

Taken together, these results highlight the importance of choline in early life for optimal brain development and cognition in healthy children. Despite this, choline requirements in pregnancy have not been adequately characterized and require increased awareness among the scientific community.

### 2.1 Insights into choline metabolism, transport, and gene regulation

#### 2.1.1 Choline metabolism

Choline metabolism is situated within the greater one-carbon metabolic pathway and is closely linked to the methionine cycle. Choline, methionine, and folate are key dietary nutrients that function as methyl donors, and their roles are closely interlinked since their metabolic pathways converge at the conversion of homocysteine to methionine. Research has shown that a disruption in one of these nutrients can trigger compensatory adjustments in the others (31). One of the few human studies investigating the metabolic relationship between choline and folate indicates that, in adults, choline serves as a methyl donor when folate levels are insufficient. This study further suggests that endogenous synthesis of choline alone does not sustain adequate levels of both choline and folate in plasma if they are under-consumed (32). In animal models, particularly pregnant mice, maternal diets rich in choline can partially mitigate the effects of folate deficiency, though not entirely (33), underscoring the unique and non-substitutable roles these nutrients play. Our manuscript will focus on the maternal choline dietary intake.

In the cell, choline can be phosphorylated by choline kinase (CK) to form phosphocholine, or it can be oxidized by choline dehydrogenase (CHDH) to form betaine aldehyde (34, 35). Phosphocholine (PC) is the precursor for the Kennedy pathway, where short-to-medium-chain phosphatidylcholines (PtdCho) are synthesized and incorporated into cell membranes (34, 36). Both lysophosphatidylcholine (LPC) and PC mediate the transport of docosahexaenoic acid (DHA) and eicosatetraenoic acid (EPA) across the blood–brain barrier via the Mfsd2a transporter (37, 38). These choline species not only support brain functionality and membrane integrity of neurons and glial cells but also facilitate the transport of DHA, which regulates the blood–brain barrier permeability (39).

Choline also serves as a precursor to sphingomyelin (SM), a crucial component of cell membranes. SM is synthesized by transferring a PC group from PtdCho to ceramide (40). During development, SM is particularly abundant due to its role in insulating neuronal axons, and it is one of the major choline compounds found in human milk (41). A recent study in humans revealed that higher levels of SM are significantly associated with improved verbal development during the first 2 years of life (42), likely due to enhanced axonal myelination.

#### 2.1.2 Choline transport

Choline enters the brain through several transport modalities, including free choline transporters: CTL1, CTL2, and CHT1 (41, 42). CTL1 is a Na + independent choline transporter, which is present in astrocytes, cortical neurons, and the endothelial cells that constitute the blood–brain barrier (43, 44). In cases of CTL1 dysfunction, CTL2 provides additional support to maintain brain choline levels (45). Recently, a novel choline transporter, FLVCR2 (also known as SLC49A2), was identified in humans. It is highly expressed in endothelial cells at the blood–brain barrier and serves as one of the primary transporters of choline into the brain (46). However, more research is needed to fully understand the regulation of FLVCR2 in response to choline availability and its potential implications for neurodevelopment.

These brain transporters are crucial in the context of maternal nutrition, choline metabolism, and embryonic neurogenesis. Once choline is transported into cholinergic neurons, it is used to synthesize ACh via choline acetyltransferase and then stored in synaptic vesicles through the vesicular Ach transporter, ready for release. In mice, the development of the cholinergic neurons begins around embryonic day (E11.5) and continues postnatally until approximately postnatal day 30 (P30) (47). During this period, CHT1 expression increases in most regions of the brain (48). Interestingly, this critical window of development is where we have demonstrated that low choline can have long-lasting effects that persist into adulthood (49). This merits more in-depth research into the long-term consequences of prenatal low choline availability on cholinergic function in both childhood and adulthood.

#### 2.1.3 Choline and its role in epigenetic regulation

In one-carbon metabolism, choline is converted into betaine, which then intersects with the methionine cycle as a methyl donor. Betaine acts as a substrate for betaine–homocysteine methyltransferase (BHMT), which remethylates homocysteine (Hcy) to form methionine (50). Methionine is subsequently used to generate S-adenosylmethionine (AdoMet), a key molecule for methylation reactions, including DNA and histone methylation. We demonstrated that during neurogenesis, low choline availability reduces methylation potential in embryonic brains (E17.5), leading to abnormal microRNA expression in neural progenitor cells (NPCs) (29). Specifically, we observed phenotypical changes in the cerebral cortex associated with the aberrant expression of miR-129p, miR-466 k, and miR-137 in NPCs under conditions of low choline availability (29). In addition, low choline intake induces CpG island hypomethylation, which results in decreased global histone methylation in hippocampal-derived murine NPCs (51). These epigenetic alterations were linked to changes in G9a histone methyltransferase activity and impacted the expression of calbindin 1, a protein involved in long-term potentiation and learning (51). In the developing cerebral cortex, the master transcription factor SRY-related HMG box 4 (SOX4) is reduced under low choline conditions. This reduction leads to decreased protein levels of its downstream target, enhancer of zeste homolog 2 (EZH2) (52). EZH2, a catalytic subunit of polycomb repressive complex 2 (PRC2), functions as a methyltransferase that tri-methylates lysine 27 of histone 3 (H3K27me3) (52).

Concurrent with the reduction in protein levels of SOX4 and EZH2, miR-129-5p has increased expression in the cerebral cortex with low choline availability (29, 52). miR-129-5p targets SOX4, leading to its reduced translation, suggesting a feedback loop in which increased miR-129-5p reduces SOX4 protein expression resulting in a lack of EZH2 transcription (52). Consequently, a reduction in total H3K27me3 was found in embryonic cerebral cortices with low choline availability compared to those with more choline availability, demonstrating the downstream effects of decreased EZH2 expression (52).

### 2.2 The role of choline in neurodevelopment: from DNA methylation to synaptic plasticity in the developing brain

The developing central nervous system is highly sensitive to nutrient availability, particularly nutrients involved in one-carbon metabolism, such as B vitamins, choline, and betaine (53). As mentioned above, one-carbon metabolism supports critical processes such DNA methylation, which regulates gene expression during brain growth and cell differentiation (54, 55). It also provides essential methyl groups for the synthesis of neurotransmitters and cell membrane components (56, 57). However, understanding the mechanisms behind behavior associated with maternal low choline status has been challenging. Fluctuations in choline dietary intake lead to changes in DNA methylation of growth-related genes such as IGF-2 and H19 (4). Furthermore, choline deficiency is linked to altered long-term potentiation (LTP) in the hippocampus (30, 58), a key mechanism for synaptic plasticity that is crucial for learning and memory (59).

In rodents, prenatal choline supplementation during neurogenesis reduces the stimulus required to elicit LTP in the hippocampus of young rodents (3–4-month-old rodents) (58). Remarkably, this increased capacity to elicit LTP in the hippocampus was found to persist far into the life cycle of the rodents (6–8- and 12–14-month-old rodents) (30). This effect is thought to be partly due to increased concentrations of Ach, which provides a larger pool of Ach in cholinergic neurons (60–62). Consequently, this could improve memory via increased synaptic plasticity in the hippocampus.

Although changes in ACh and LTP have helped to explain part of the behavioral changes related to choline availability, gaps remain in fully understanding how choline impacts cognitive function. Emerging studies are shedding light on structural changes in the brain. In the developing hippocampus of embryonic rodents under low choline availability, progenitor cell proliferation was found to be reduced, and the number of progenitor cells undergoing apoptosis increased, compared to the hippocampi of embryonic rodents with higher choline availability (63).

Moreover, angiogenesis in the hippocampus was found to be impaired in embryonic rodents exposed to low choline availability compared to those with high choline availability (64). Similar effects were observed in the cerebral cortex. Embryonic mice exposed to low choline availability during E11.5–E17.5 were found to have a substantially reduced pool of NPCs and increased apoptosis in the cerebral cortex compared to embryonic rodents with higher choline availability (49). In addition to the reduced pool of NPCs, it was also observed that there were altered proportions of cortical early and late born neurons resulting in aberrant cortical layering, characterized by a reduction of pyramidal neurons (49). This aberrant cortical layering was found to persist later in life (4-month-old mice) (49). The changes in neuronal populations are partly driven by a reduction in the epidermal growth factor receptor (EGFR) protein levels due to low choline availability, leading to a reduction in the proliferation of NPCs (49). These findings may help explain suboptimal neurodevelopment in offspring exposed to inadequate maternal choline intake during pregnancy.

Abnormal corticogenesis using genetic tools to alter neurogenesis has been shown to impact sensorimotor integration and neocortex-dependent long-term memory recall, particularly in tasks such as contextual fear conditioning (65). Moreover, neurons in layer 2/3 of the cerebral cortex play a crucial role in controlling behavioral responses to visual disturbances (66). Given that choline influences both cortical development (49) and retinogenesis (28), we hypothesize that changes in choline availability may contribute to altered behavioral phenotypes. This hypothesis is further supported by findings from a knockout model of the choline pathway enzyme, betaine–homocysteine methyltransferase (Bhmt), in which mice exhibit reduced brain volume and impaired memory function (67).

## 3 Choline and neurodevelopmental disorders with a known genetic origin

Although we have a solid understanding of the impact of choline availability on neurodevelopment in a non-diseased state, an outstanding question is the impact of choline availability on the manifestation and symptomology in different neurodevelopmental disorders. In this study, we will focus on Rett syndrome, fragile X syndrome, and Down syndrome, highlighting the clinical characteristics, their underlying molecular mechanisms, and the potential beneficial effects of adequate choline intake.

### 3.1 Rett syndrome

#### 3.1.1 Clinical characteristics of RTT

Rett syndrome is a complex neurodevelopmental disorder predominantly affecting female individuals, characterized by severe cognitive and physical impairments. Initially, children with RTT exhibit apparently typical development, followed by a progressive loss of fine and gross motor skills, deceleration of head growth, and repetitive hand movements. Diagnosis of RTT includes identifying abnormal breathing patterns, such as hyperventilation or period apnea (68, 69). In the early stages, some girls may be misdiagnosed with autism spectrum disorder until the hallmark motor skill deterioration becomes apparent, prompting a reevaluation of the clinical case.

#### 3.1.2 The underlying molecular mechanism of RTT

Rett syndrome is an X-linked neurodevelopmental disorder linked to mutations in the *MECP2* gene located on the X chromosome (70). This gene encodes methyl-CpG binding protein 2 (MeCP2), a critical protein for brain development, and is predominantly expressed in neurons after embryonic neurogenesis has concluded (71). MeCP2 contains a methyl-CpG-binding domain (MBD), which binds to chromosomes in a methylation-dependent manner (72–75). MeCP2 recruits other corepressors, through a transcriptional-repression domain (TRD), including the SIN3A histone deacetylase (HDAC) complex and NCoR/SMRT co-repressor complexes (72, 76). This process is essential for preserving chromatin structure and consequently gene expression (77, 78).

Methyl-CpG binding protein 2 is localized on chromatin and can bind to both the nucleosome complex and methylated DNA (79). DNA methylation not only can directly control MeCP2 expression but also can function. In a mouse model, locus-specific DNA methylation of the *Mecp2* promoter led to autism-like behaviors (80). The localization of MeCP2 to chromocenters is suggested to be primarily determined by global DNA methylation patterns, independent of heterochromatin foci and phase separation (81). Although MeCP2 binding to DNA persists in the absence of DNA methylation, this binding is promiscuous and transient, suggesting that DNA methylation is the key factor in MeCP2 binding.

#### 3.1.3 Choline and potential interactions with RTT

Low choline availability results in the alteration of global CpG island methylation in the developing brain (54, 82). Methylation of CpGs is carried out *de novo* by DNA methyltransferase 3A, with stabilization by DNA methyltransferase 3 L (DNMT3L) and 3B (DNMT 3A/3B). DNA methyltransferase 1 (DNMT1) maintains these marks through cell division, a process supported in part by DNMT3L (83). In the hippocampus, low choline availability resulted in CpG hypomethylation, leading to the increased expression of several genes including kinase-associated phosphatase, calretinin, and cyclin-dependent kinase inhibitor 2B. Conversely, in the cortex, 5-methylcytosine levels are elevated (82). This hypermethylation is linked to the increased expression of several DNMTs, including DNMT1, DNMT3a, and DNMT3L, which contributes to the aberrant expression of several genes including insulin-like growth factor 2, G9a, and calbindin 1 (51). MeCP2 preferentially binds to methyl-CG dinucleotides, predominantly CpG islands, which are adjacent to regions enriched in adenine (A) and thymine (T) bases (84).

In addition to its effects on CpG islands, choline also influences histone methylation. Low choline levels reduce methylation at the H3K27me3, as well as at H3K9me2 and H3K9me1. Methylation of H3K9 and H3K27 is critical for the formation of heterochromatin as it recruits and maintains heterochromatin protein 1α through PRC2-mediated cooperation. MeCP2 was found to bind with histones; however, binding was increased in the presence of H3K27me3 (85). It is suggested that H3K27me3 helps to recruit MeCP2 to facilitate gene repression as *Mecp2* knockout mice have increased expression of genes that are regulated by H3K27me3 (85). Interestingly, our research, as mentioned above, shows that choline availability alters the methylation levels of H3K27me3; potentially, choline can play an indirect role in the recruitment of MeCP2 (81). Moreover, modified variants of MeCP2 that bind only to methyl-CpGs were found to be insufficient in maintaining neurological function (86). Taken together, these findings suggest that choline availability may influence MeCP2’s role in gene regulation through its effects on methylation and chromatin structure.

Despite the genetic origin of RTT, encouraging outcomes have been published (87), using an *in vitro* model of choline supplementation. In this model, the expression of the MeCP2 protein was knocked down with shRNA, leading to improvements in synaptic function and behavior. Furthermore, a study using a Rett syndrome mouse model (i.e., Mecp2-null) demonstrated that postnatal choline supplementation attenuated some behavioral deficits in the offspring (88). These promising findings underscore the need for further research in animals and humans to better understand the potential role of choline in alleviating RTT symptoms.

### 3.2 Fragile X syndrome

#### 3.2.1 Clinical characteristics of FXS

Fragile X syndrome is recognized as a leading cause of intellectual disability and autism spectrum disorder. Children with FXS often experience developmental delays and autism-like behaviors such as hand flapping, poor eye contact, and challenges with social interactions. In addition, behavioral alterations are common, including anxiety, delayed language milestones, and in some cases, seizures, strabismus, and obesity (89). These children meet the criteria for attention deficit hyperactivity disorder (ADHD) and autism. Typically, FXS is diagnosed around the age of three, often as a result of a follow-up assessment of recurrent developmental concerns observed during early childhood. In most cases, parents are referred to a clinical geneticist, where a blood test is conducted to detect mutation in the *FMR1* gene, which confirms the diagnosis of FXS (90, 91).

#### 3.2.2 The underlying molecular mechanism of FXS

Fragile X syndrome is caused by a reduction or complete absence of fragile X messenger ribonucleoprotein 1 (FMRP). FMRP is an RNA-binding protein that is involved in the regulation of many mRNAs within postsynaptic neurons, contributing to synaptic plasticity and brain structure. The most common cause of FXS is the expansion of the CGG trinucleotide repeat in the promoter region of the gene *FMR1* (92). This leads to methylation of the *FMR1* promoter region and subsequent transcriptional silencing. This altered epigenetic landscape occurs around the 11th week of gestation and is associated with H3 demethylation, which is mediated by the formation of DNA–RNA duplexes between the CGG repeat region of the *FMR1* promoter and its mRNA counterpart (93). Consequently, this leads to the silencing of the *FMR1* gene by FMR*1* mRNA (93).

Individuals with more than 200 CGG (full mutation) experience complete transcriptional silencing of *FMR1*, while individuals with 55–200 CGG repeats (premutation) exhibit increased transcription of *FMR1* (94). In addition, some individuals can have a mosaicism, where certain cells show methylation of the *FMR1* promoter, while others are unmethylated, leading to varying levels of FMRP expression (94). The degree of methylation of the *FMR1* promoter is correlated with a spectrum of behavioral deficits in which the severity of the disease is related to the extent of altered *FMR1* gene expression and subsequent FMRP protein levels (95).

Fragile X messenger ribonucleoprotein 1 protein is predominantly located in the cytoplasm, associated with free ribosomes within dendritic spines. In neurons, it is involved in RNA stability, splicing, and RNA interference (96). Preclinical models of FXS replicate some of the phenotypes observed in humans, contributing to our understanding of the disease and revealing potential pathways for treatment (97).

#### 3.2.3 Choline and potential interactions with FXS

Fragile X syndrome is characterized by the disruption of cholinergic function, which contributes to the behavioral and cognitive impairments observed in the disorder. A recent study using proton magnetic resonance spectroscopy explored choline concentration in the brains of male individuals with FXS. The findings revealed a significant reduction in the choline concentration and choline/creatine ratio (98). This suggests that increasing choline availability might partially restore cholinergic function. A recent study demonstrated that a combined intervention of donepezil and choline improved language abilities in children with autism (99). Interestingly, donepezil is also a recognized treatment for FXS. This evidence establishes a foundation for investigating the potential benefits of choline supplementation in combination with pharmaceutical treatment for FXS.

In a forebrain organoid model of FXS, dysregulated neurogenesis was observed, characterized by a reduced number of actively proliferating Ki67+ NPCs (100). As previously discussed, prenatal choline availability regulates NPC proliferation in the developing cortex (49). This raises the question of whether choline supplementation could enhance the reduced NPC proliferation observed in FXS. In addition, in a knockout model of FXS, a phenotype of dysregulated neuronal maturation was noted. This aligns with findings where *Fmr1* is identified as a significant regulatory target of miR-129-5p, which controls neuronal migration during cortical neurogenesis. Choline regulates miR-129-5p in the cerebral cortex, and *in utero* inhibition of miR-129-5p was able to rescue the neuronal migration defects caused by low choline (29). Further investigation into how choline influences cortical development in different FXS models would be valuable.

### 3.3 Down syndrome

#### 3.3.1 Clinical characteristics of DS

Down syndrome results from a trisomy of chromosome 21 (Hsa21), leading to the abnormal function of multiple bodily systems, including musculoskeletal, neurological, and cardiovascular systems (16). Apart from the facial phenotype, DS is characterized by impaired language and memory capacities. Understanding the pathology of DS and its impact on these systems is complex, primarily due to the triplication of over 200 protein-coding genes. Advances in human genome sequencing have enabled the identification of specific genes and their epigenetic regulation on Hsa21, shedding light on the potential consequences of this genetic alteration. For example, it is now well-established that a subset of individuals with DS are at a higher risk for early-onset Alzheimer’s disease, attributed to a dose-dependent increase in amyloidogenic fragments (101, 102). Effective management of DS requires a holistic approach, addressing psychiatric care to manage behavioral changes, as well as nutritional and metabolic health (103).

#### 3.3.2 The underlying molecular mechanism of DS

In the brains of individuals with DS, a reduction in cortical volume and a decrease in overall number of neurons have been observed (104). This finding aligns with studies in mouse models that show a reduction in the pool of NPCs in the cerebral cortex, suggesting that impaired neurogenesis may contribute to the brain phenotype observed in DS. Not only is gene expression altered, but methylation and epigenetic marks are also altered in DS (105). Interestingly, some dysregulated genes, such *DYRK1A*, play a role in this process by phosphorylating downstream proteins such as p21, which regulates the G1/G0-S cell phase transition (106). This dysregulation impacts NPC differentiation, promoting a shift toward glycogenesis, which may explain the altered proliferation and reduced number of neurons observed in DS (107).

Altered methylation patterns on HSA21 are reported in individuals with DS (108). This may be linked to a reduction in the methylation potential, as reduced levels of AdoMet have been observed in DS (109). This alteration in one-carbon metabolism, including reduced methionine levels and increased cystathionine, may contribute to the underlying causes of altered DNA methylation patterns in DS.

#### 3.3.3 Choline and potential interactions with DS

Environmental conditions, such as nutritional and choline status during critical periods of development, particularly during pregnancy and early childhood, may play a critical role in DS. Findings from animal studies have shown that prenatal choline supplementation can improve attention and reduce cognitive and affective dysfunctions in the Ts65Dn mouse model of DS (110). In addition, maternal choline supplementation has been shown to enhance hippocampal neurogenesis in the offspring of the Ts65Dn mouse model, compared to the non-supplemented groups. The improvement in hippocampal neurogenesis was directly correlated with coordination and performance, suggesting that an improvement in neurogenesis causes better cognitive special performance (111).

The relevance of maternal choline intake extends beyond early development as studies in adult wild-type rats have demonstrated its positive impact on neurogenic responses, indicating long-lasting effects on adult neurogenesis (112). It is likely that the mechanism by which choline supplementation exerts its effects in Ts65dn mice is also through enhanced neurogenesis. Choline, via its metabolite betaine, influences methylation potential and, therefore, regulates gene expression. Specifically, in the Ts65Dn mouse, maternal choline supplementation produced long-term effects on gene expression in pyramidal neurons. Interestingly, GABAergic genes were upregulated in response to choline, which could explain the observed improvements in learning and memory (113).

Moreover, a case–control study revealed that in humans, genetic variants of the *BHMT* gene (rs3733890) increase the risk of DS, particularly in populations with folate impairment (114). Although larger studies are needed, these findings highlight the importance of considering genetic factors, as well as the potential of choline and its metabolites, in the context of DS risk.

### 3.4 Gaps and future directions

Choline and its metabolites are important in the developing brain, and disruption of proliferation and differentiation pathways during fetal development can result in substantial brain structure alterations. However, it remains an open question whether choline availability can ameliorate the symptoms of neurodevelopmental disorders.

A major gap in the field is the need to incorporate genetic factors into dietary intake recommendations for humans. While there is substantial evidence demonstrating the benefits of choline during brain development, the majority of studies have been conducted in animal models such as mice and rats. Although the mechanistic basis of choline’s beneficial effects is not fully understood, choline and its metabolites may influence the brain environment through gene regulation, cell signaling, and neurotransmission.

Improvements in symptomology in RTT and FXS in response to increased choline intake suggest that choline may have also impact X-linked diseases by ensuring sufficient AdoMet concentrations, which are essential for X chromosome inactivation (XCI). XCI is critical for maintaining X-linked dosage compensation between male and female cells and for the survival of female embryos. Choline’s control on two specific histone marks, namely, H3K9 and H3K27, may change how key silencing complexes such as PRC2 and chromodomain Y-like protein (CDYL)-G9A-MAX gene-associated (MGA) are recruited during later stages of XCI (115). This altered recruitment could potentially perturb downstream XCI processes including XCI maintenance. Finally, exploring the role of choline supplementation, both alone and in combination with other drugs, in neurodevelopmental disorders is a promising area of research.

## 4 Conclusion

Emerging studies continue to highlight the critical role of choline in brain and nervous system development. Yet, precise biomarkers for choline status, particularly in pregnant women, remain elusive. In the US, women of reproductive age fail to meet the recommended choline intake. Thus, it is imperative to reassess the recommended choline intake during pregnancy, taking the genetic factors of the fetus and mother into consideration. In addition, further research is warranted to explore the impact of choline on the maternal gut microbiome and its potential influence on the developing brain. With the rising incidence of neurodevelopmental disorders in the US and globally (116) and the displacement of nutrient dense foods by ultra-processed alternatives, addressing choline intake is more crucial than ever (117). In animal models, choline supplementation during pregnancy is both safe and cost-effective, but more studies are needed to elucidate the molecular mechanisms through which it may protect against neurodevelopmental disorders.
